# Lab-in-Syringe Automated Miniaturized Bioconjugation of Magnetic Beads with Anti-SARS-CoV2 Antibodies

**DOI:** 10.1021/acsomega.4c11126

**Published:** 2025-07-21

**Authors:** Zuzana Svobodova, Lucie Krizova, Nikola Matejkova, Denisa Smela, Martin Beranek, Zuzana Bilkova, Burkhard Horstkotte

**Affiliations:** † 37740 Charles University, Faculty of Pharmacy, Department of Biological and Medical Sciences, Hradec Kralove 500 09, Czech Republic; ‡ 37740 Charles University, Faculty of Pharmacy, Department of Analytical Chemistry, Hradec Kralove 500 03, Czech Republic; § 48252 University of Pardubice, Faculty of Chemical Technology, Department of Biological and Biochemical Sciences, Pardubice 532 10, Czech Republic; ∥ Institute of Clinical Biochemistry and Diagnostics, Charles University Hospital and Faculty of Medicine in Hradec Kralove, Hradec Kralove 500 03, Czech Republic; ⊥ 37740 Charles University, Faculty of Pharmacy, Department of Biochemical Sciences, Hradec Kralove 500 03, Czech Republic

## Abstract

We present the first automated synthesis of magnetic immunosorbents (MIS) using a lab-in-syringe (LIS) platform, facilitating antibody bioconjugation to magnetic beads via carbodiimide-mediated covalent binding. This approach is an efficient, reproducible alternative to traditional manual methods, minimizing pipetting steps, vortexing, and incubation with a reduced handling bias. Utilizing a 1 mL syringe pump with a 12-port multiposition valve and an internal magnetic stir bar enables precise mixing, bead dispersion, and magnetic capture for consistent bioconjugate synthesis. The LIS platform achieved a 99.6% bead recovery with 0.4 mg of MIS (1 μm in diameter), outperforming the 83% recovery of manual techniques, and maintained an 83% recovery at reduced scales of 0.2 mg, surpassing manual yields of 76%. As a proof-of-concept, MIS conjugated with anti-SARS-CoV2 antibodies (6 μg/400 mg beads) were synthesized and validated for viral RNA isolation from COVID-19-positive samples, demonstrating high immunocapture efficiency comparable to manual methods but with significantly reduced time and labor requirements. This automated synthesis of antibody-MIS enables the scalable, reproducible production of bioconjugated materials, supporting advanced applications in diagnostic assays, therapeutic delivery, and microfluidic integrations. The LIS approach thus enhances the scope of biomolecular conjugate synthesis, offering streamlined workflows that are suited for downstream analytical and bioanalytical applications. LIS is a versatile, automated system for preparing MIS that researchers can adapt for various targets, particularly when commercial products are unavailable, are prohibitively expensive, or require custom carriers.

## Graphical abstract

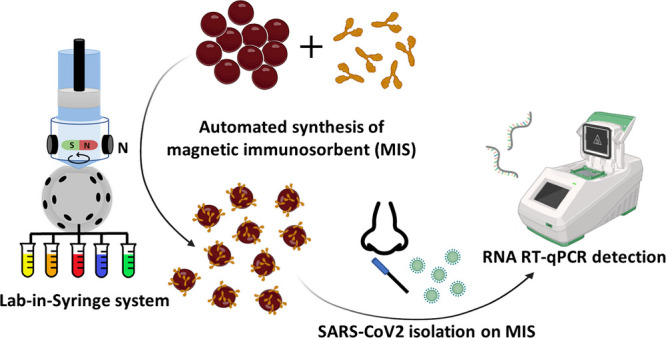

## Introduction

Magnetic micro- and nanobeads (MBs) have received considerable attention as supports for biomolecular conjugates due to their easy handling, large ratio of surface area to volume, low cost and toxicity, and compatibility with biomaterials. These properties make MBs versatile tools for bioconjugation, functioning as carriers for biomolecules, reaction support phases, and tools for target separation. MBs are extensively used in various areas of basic or clinical research, with applications ranging from magnetic hyperthermia^1,2^ and targeted drug delivery^3,4^ to tissue engineering,^5^ and the magnetic separation of biological objects such as cells,^6^ bacteria,^7,8^ viruses,^9^ exosomes,^10^ DNA,^11^ and proteins.^12,13^ MBs also play a critical role in diagnostic immunoassays,^14,15^ making them valuable tools in bioconjugation for immunoaffinity separations, where the specific antigen–antibody interaction is exploited for target capture, enrichment, and detection.^16^

The most often mentioned applications of MBs bioconjugated with antibody or antigen are magnetic ELISA,^17^ magnetic CLIA,^18^ microfluidic platforms (centrifugal,^19,20^ pump-related,^21,22^ digital microfluidics^23,24^ ), and manual-batch techniques.^25–27^

Magnetic immunosorbents (MIS), which consist of antibody-coated MBs, can either be prepared in the laboratory or purchased as ready-to-use materials. However, commercially available MIS products are often limited in terms of size, material, and specificity and are typically sold in large bulk volumes. This restricts their use in research where smaller customized batches are often required.

The in-laboratory bioconjugation process, referred to here as the manual procedure, is typically carried out by an operator using a tube and involves multiple manual steps, including pipetting, vortexing, and incubation in a magnetic stand or shaker. MBs are available in various sizes, from tens of nanometers to hundreds of micrometers, and with diverse surface functionalities, including amine, carboxyl, epoxy, and hydroxyl groups, among others.^28^ Common methods of bioconjugation involve either noncovalent, via biologically active molecules such as biotin, streptavidin, and protein A/G, or covalent bonding via cross-linkers,^16^ such as the carbodiimide approach (using N-(3-(dimethylamino)­propyl)-N′-ethylcarbodiimide (EDC) and sodium N-hydroxysulfosuccinimide (S-NHS) for carboxyl group activation), being the most widely used for covalent nonoriented attachment.^29^ The manual process offers flexibility in bead size, surface functionalization, and antibody specificity. Still, manual synthesis remains labor-intensive and prone to batch-to-batch variability, posing challenges to reproducibility and loss of material (MIS) due to repetitive handling. In response to these challenges, there has been growing interest in automating bioconjugation procedures, particularly through flow chemistry, which ensures precise control over reaction conditions, reproducibility, and efficiency. Automation not only minimizes handling errors and contamination risks but also enhances the yield and quality of the final bioconjugates by optimizing mixing and timing conditions, leading to efficient utilization of reagents, minimization of waste, and the need for consumables compared to the manual procedure.

Here, we present the first automated system for the synthesis of magnetic immunosorbents (MIS) using a lab-in-syringe (LIS) platform. This system leverages carbodiimide chemistry for covalent antibody attachment and provides a highly reproducible alternative to traditional manual methods. The LIS technique involves the use of an automatic syringe pump equipped with a 12-port multiposition valve, with a magnetic stir bar inside the syringe to ensure homogeneous mixing and efficient bead capture,^30^ see Figure 1.

**Figure 1:**
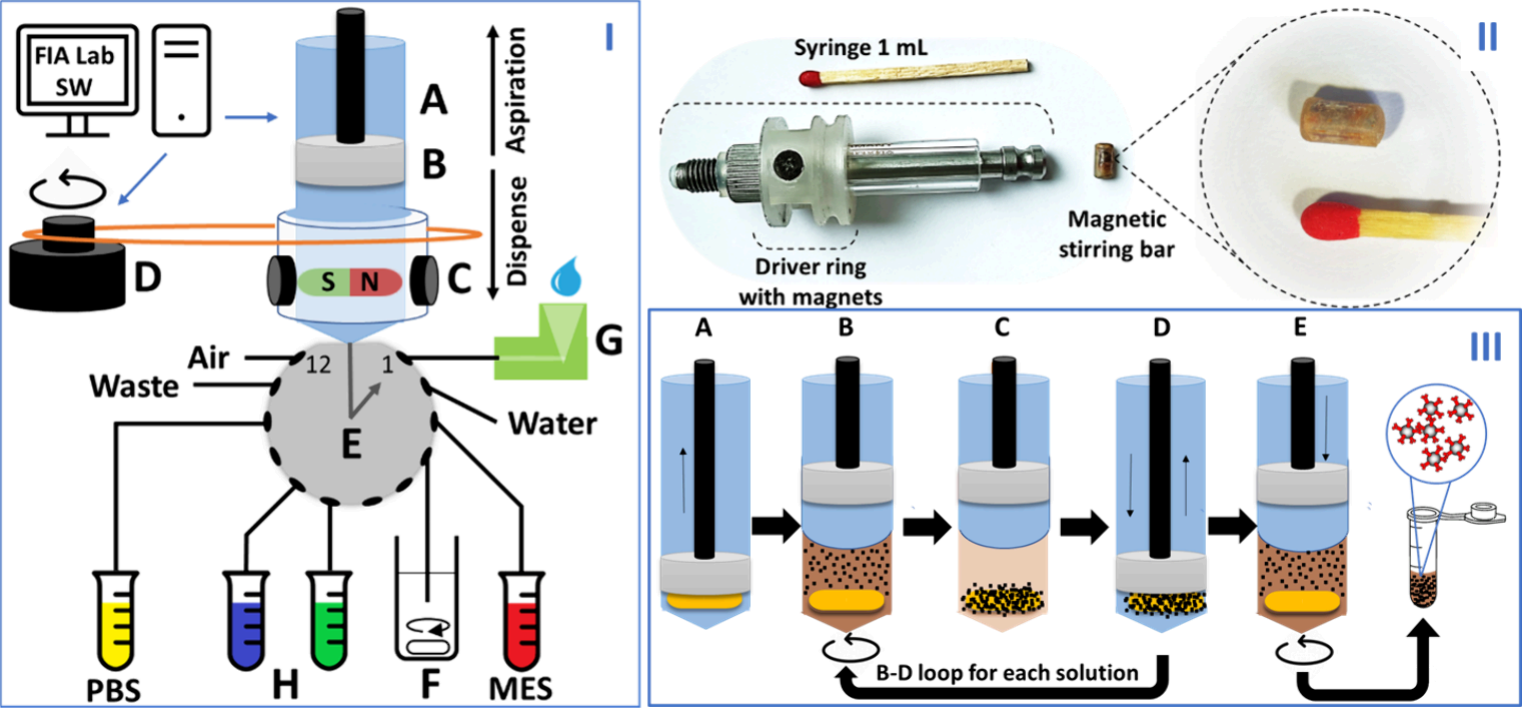
I: LIS system: A, computer-controlled glass syringe pump to aspirate and dispense liquids from 12-multiposition head valve; B, Teflon piston; C, magnetic stir bar and driver ring with two magnets enabling mixing of solution or beads; D, motor connected by a rubber ring to driver ring enabling rotation of the stir bar inside the syringe (computer controlled); E, 12-port multiposition head valve; F, MBs suspension, G; open port interface (OPI) for antibodies and activation reagents; H, vials for IgG coated MBs collection. II: Photography of a 1 mL syringe with a driver ring and laboratory-made stir bar. III: Scheme of procedural steps: A, solution/MBs uptake; B, stir bar spins and mix the MBs during incubation or washing step; C, MBs attracted to magnetic stir bar; D, solution discharge/aspiration (beads remain inside the syringe); E, discharge of dispersed MBs coated with antispike IgG into a collection vial, involving simultaneous stirring and dispense of the bioconjugated beads suspension (repeated 4 times).

Previous studies^31,32^ have demonstrated the versatility of this tool in liquid-phase microextractions and solid-phase extractions,^33–35^ and here we extend its application to the automated bioconjugation of antibodies onto MBs. The entire bioconjugation procedure involves sequential MBs washing, their activation with EDC and S-NHS, incubation with the antibody (a covalent bond is created between activated MBs and lysine-containing antibodies), and final washing before resuspension in a storage buffer. The automated bioconjugation system offers significant advantages for the reproducible, on-demand synthesis of bioconjugated magnetic beads, providing a streamlined workflow that reduces both time and labor.

The model system with SARS-CoV2 was used to demonstrate the proof-of-concept for antibody bioconjugation to magnetic beads by using the LIS technique. Various detection methods for SARS-CoV2 have been developed, including paper-based assays,^36^ PCR-based protocols,^37^ and fluorescence-based approaches.^38,39^ In this study, we employed standard reverse transcription quantitative PCR (RT-qPCR) to detect SARS-CoV2 RNA following immunomagnetic isolation targeting the spike protein expressed on the surface of the viral particle. Following immunocapture, the viral particles were lysed and the extracted RNA was analyzed via RT-qPCR. The use of COVID-19-positive human samples enabled us to validate that viral particles can be efficiently and quantitatively captured using MIS prepared either manually or via the automated LIS method.

## Results and Discussion

### Automation of Antibody Bioconjugation to Magnetic Beads and Aimed Innovation

Flow techniques have been repeatedly used for the handling of immunosorbents. Most recently, mesofluidic automation has been employed for the noncovalent bioconjugation of IgG to protein A-coated agarose beads within a microcolumn. For this, the loaded mass of human IgG typically ranged from 0.1 to 0.4 μg per 5.5 mg of agarose beads (34 μm in diameter), and the immobilized IgG was detected in situ on the Lab-On-Valve platform.^40^ However, to the best of our knowledge, preparation of MIS using the carbodiimide method has not yet been reported in either flow or flow-batch systems, and not in such a low amount of MBs (0.2 mg). Moreover, the LIS system offers efficient mixing and repetitive incubation of MBs with various reagents.

Herein, we report an automated method for the covalent bioconjugation of MBs and ligands via carbodiimide chemistry, which is a commonly used methodology for the conjugation of carboxy groups on MBs and to primary amine groups (e.g., on arginine or lysine residues) of antibodies. In this reaction, EDC activates the carboxyl groups on the MB surface, forming an unstable O-acylisourea intermediate. To improve stability and reactivity, NHS is added to convert the intermediate into a more stable NHS ester, which readily reacts with primary amines (such as those on lysine side chains of IgG molecules), forming stable amide bonds and releasing NHS and urea byproducts.^41^

The antispike antibody was selected for viral particle isolation due to the surface presentation of the spike protein on SARS-CoV-2 virions, which ensures high accessibility for binding. Additionally, the spike protein’s receptor-binding domain contains unique sequences that distinguish SARS-CoV2 from other coronaviruses, making it ideal for specific capture. Following immunocapture, the viral particles were lysed, and the extracted RNA was analyzed via RT-qPCR.

Concerning the size, MBs of ca. 1 μm diameter were chosen over nanoscale beads to ensure a sufficiently strong magnetic susceptibility and thus the efficient capture of the beads on the magnetic stir bar. In follow-up studies, we plan to study the use of smaller MBs to benefit from a higher surface area/volume ratio and achieve a higher analyte recovery rate.

While the manual bioconjugation procedure consists of repeated manual steps, foremost vial manipulation, bead capture by the magnetic separator, solution switching, pipetting, or discarding, bead suspending by vortexing after each magnetic separation step, and vial placement in the rotator for lasting incubations. In the LIS system, the main advantage is an all-in-one concept. The syringe void simultaneously serves as a vial, pipet, and rotator; the stir bar inside is a magnetic separator and vortex mixer (Figure 1, III).

During incubation steps, such as bead activation and antibody bioconjugation, continuous mixing or rotation was replaced by intermittent stirring at 1500 rpm, with 3 s of stirring followed by a 12 s pause, minimizing mechanical stress by protecting the beads from shearing forces. The stirring speed was studied in the range of 400–1700 rpm (see photos in Figure 2). Visual inspection confirmed that all MBs overcame the magnetic attraction of the stir bar at 1500 rpm, which was selected as the optimal speed—the minimum required for effective MB resuspension while minimizing shear forces. Additionally, LIS brings benefits in that all steps can be optimized one by one and transformed into the software programming language commands (see Supporting Information Table S-1 and S-2).

**Figure 2:**
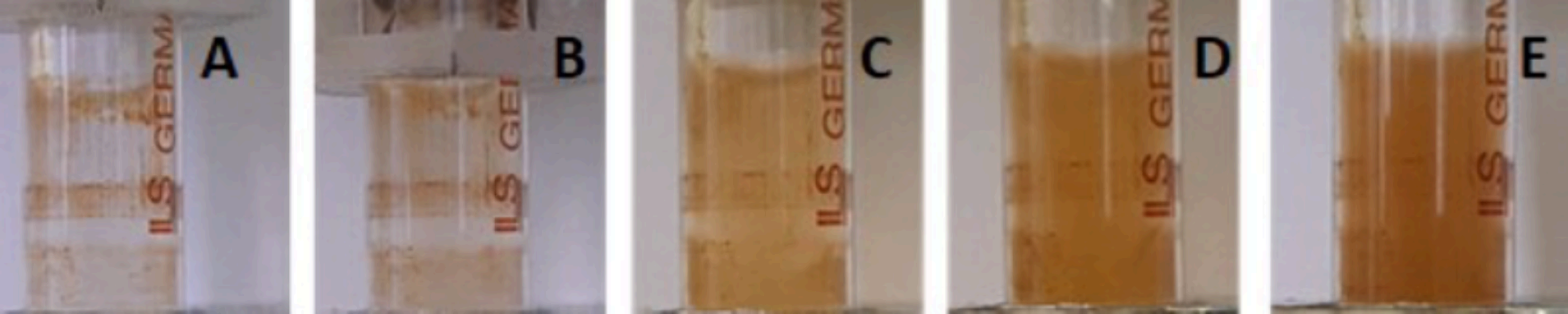
Photographs of the syringe void during the steering speed study: (A) 0 rpm, (B) 400 rpm, (C) 750, (D) 1100 rpm, and (E) 1500 rpm. The speed of 1700 rpm was also tested but data are not shown, and the color density was the same as at 1500 rpm. The speed of 1500 rpm was selected for the experiments as it is the minimal speed when all MBs are resuspended.

The only manual step required was the pipetting of freshly prepared activation reagents and antibody solution into the open port interface (OPI, Figure S-1.III and Figure S-2.II in Supporting Information). Emptying the open port interface and cleaning, if needed, were done in an automated fashion (see Experimental Procedures, LIS-Automated Bioconjugation of Antibodies to MBs section). These steps could be automated by combining the LIS system with an autosampler with vial cooling to achieve truly stand-alone MIS synthesis.

All steps carried out by the automated LIS system were controlled by a PC using FIALab software and were thoroughly tuned, tested, and optimized as described in the Supporting Information (Section S1.2, supplemented by Figures S-3 to S-5 and Tables S-3 to S-4).

The bioconjugation process in the LIS system begins by aspirating MBs from a glass vial containing the MB suspension (Figure 3). After an initial washing step, the MBs are activated by adding the carbodiimide reagents EDC and S-NHS via the open port interface (OPI; 200 μL volume) and incubated for 15 min under intermittent stirring. Following reagent removal and a washing step, the antibody is introduced through the OPI and incubated for 1 h under intermittent stirring. After a final washing step, the resulting magnetic immunosorbents (MIS) are collected into vials using simultaneous stirring and syringe dispensing; for further details see Experimental Procedures and Supporting Information, Table S1).

**Figure 3:**
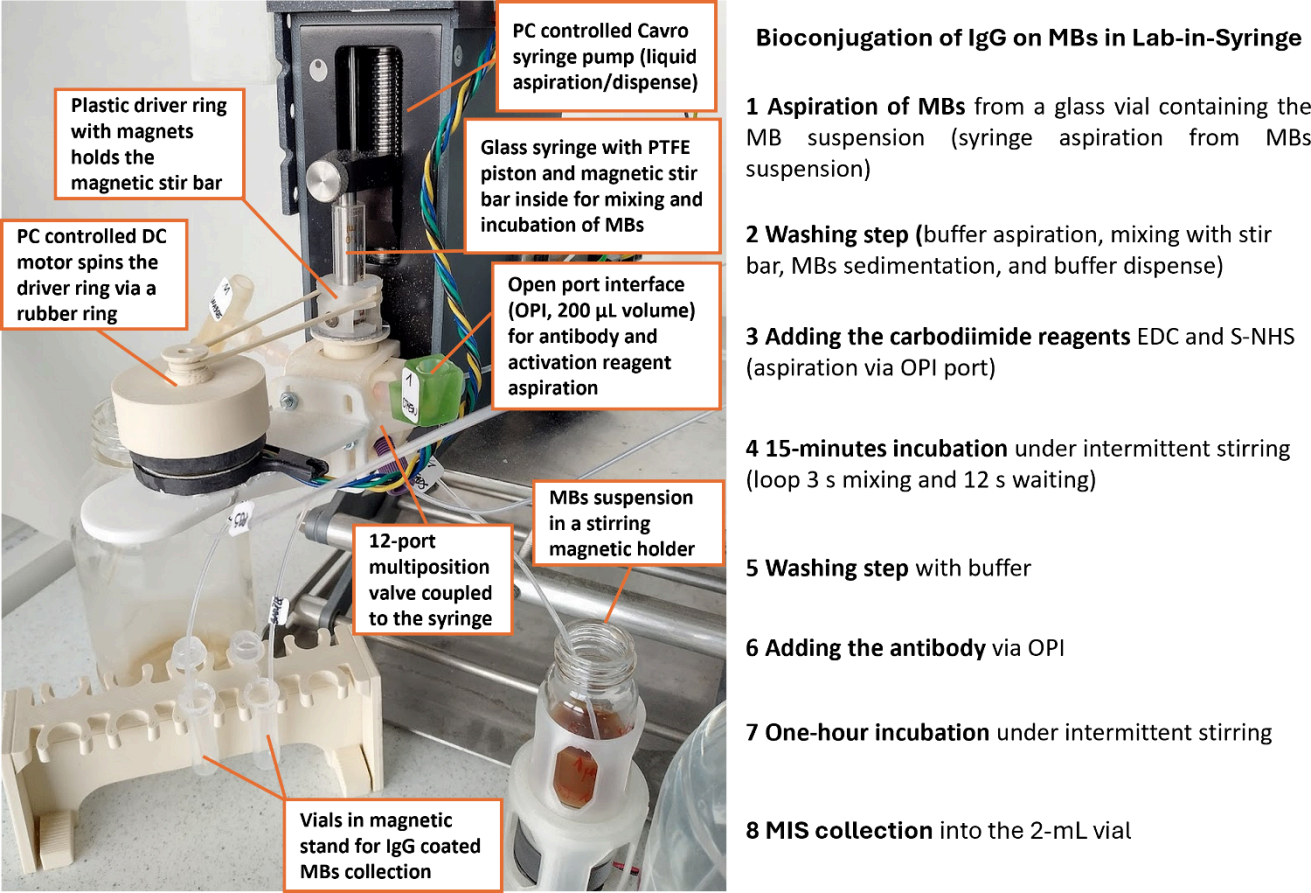
Photograph of the automated Lab-in-Syringe (LIS) system used for the covalent bioconjugation of antibodies to magnetic beads (MBs) via the carbodiimide method. For additional details, see Experimental Procedures and Supporting Information, Table S1 and Figure S-1.

### Enhanced Magnetic Beads Recovery in the LIS Automated MIS Synthesis Procedure

Automation aims to enhance the consistency of procedural execution and operator dependability by reducing handling variability or even liberating human resources. Consequently, it typically leads to an increase in the accuracy and precision. Moreover, automation frequently accelerates task completion compared to manual methods, thus increasing the procedural and cost efficiency. To prove such a yield in performance, we critically compared both procedures regarding MB collection recovery after the procedure completion and handling precision.

The optimal amount of MBs for the LIS-automated synthesis procedure regarding collection recovery of MIS was studied in the range of 0.2 to 1.0 mg (Supporting Information Figures S-9, S-10). Aiming for miniaturization of the invested sorbent, the loads 0.2 mg and 0.4 mg of MBs were employed for further experiments. Both the manual and the automated procedures were carried out in triplicate and we evaluated bead recovery, handling precision, and in particular, the loss of beads.

The loads of 0.4 and 0.2 mg of MBs were applied into the vials for manual synthesis, LIS synthesis, and controls in triplicates. Manual syntheses were performed in a 1 mL volume using 2 mL Eppendorf vials, while LIS syntheses were conducted in a 1 mL syringe, as outlined in Experimental Procedures. However, instead of activation reagents and antibodies, PBS buffer was used. Controls of the initial MB suspension were washed once with PBS. The MBs from manual and LIS syntheses were collected into Eppendorf tubes (2 mL), and the absorbance of the bead suspensions was measured at 600 nm as well as the controls.

The results given in Figure 4 showed that the LIS-automated synthesis procedure provided a higher recovery (83 ± 5%) using 0.2 mg of MBs compared to the manual protocol (76 ± 6%). The results obtained with an initial amount of 0.4 mg MBs showed an even higher yield of 99.6 ± 0.7% bead recovery versus 80.8 ± 14% for manual bioconjugation. Thus, the LIS protocol provided results with higher recovery and lower standard deviation, in other words, improved precision and product yield.

**Figure 4:**
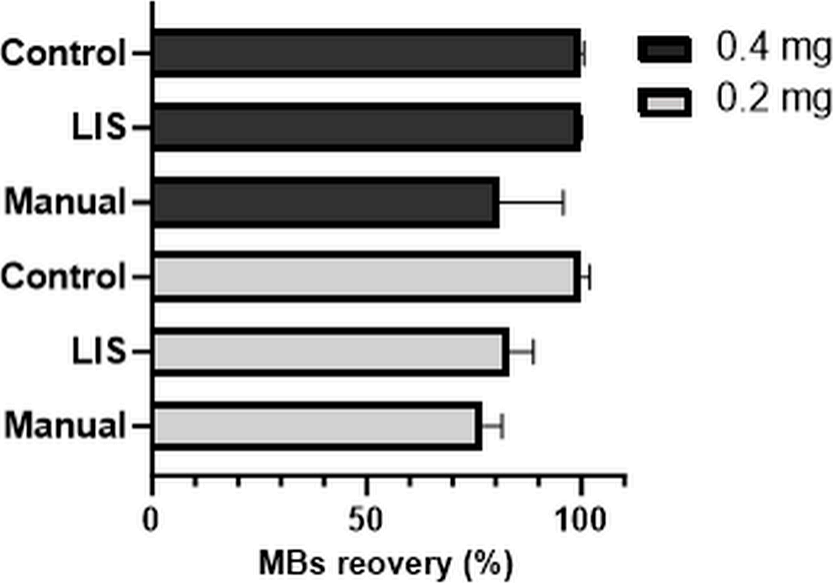
Recoveries of repetitive bioconjugation of MBs in triplicate performed manually and automated with LIS were compared with the control aliquot of MBs that did not undergo the bioconjugation procedure. Two amounts of MBs were tested (0.2 mg and 0.4 mg of MBs). The amount of MBs was evaluated by spectrophotometric measurements at 600 nm (see Supporting Information, Figure S-8).

This suggests that lower amounts of MIS can be prepared reliably by LIS automation, making the approach highly useful for bead bioconjugation with ligands of high costs or difficult-to-come-by ligands or MBs. It should be noted that the outcomes of the experiments depend on the magnetic strength of the stir bar, driver ring, and syringe diameter.

In addition, preliminary experiments were conducted to upscale the automated procedure to a 5 mL syringe and increase the size and force of the magnetic stir bar. It was shown that near-to-quantitative recovery using up to 20 mg MBs is possible with 1.2–1.7% (0.17–0.34 mg) MBs leaking per washing cycle (see Supporting Information, Figure S-11). Consequently, the working volume, amount, and type of MBs and bioconjugation technique are considered adaptable according to the intended application. Plus, the method optimization of the procedural parameters is easily achievable.

### LIS-Synthesized MIS in SARS-CoV2 RNA Isolation from COVID-19-Positive Samples

Anti-SARS-CoV2 MISs, with the ability to isolate viral particles from nasopharyngeal samples, were prepared by both automated LIS and manual methods to determine whether we can achieve comparable results and thus demonstrate the proof of concept. In the manual protocol, an incubation for 2 h on the rotator was used. In LIS automation, the risk of MBs being damaged due to the long stirring that might cause shearing forces must be considered. Therefore, we applied the shortening of the incubation time for antibody conjugation to 10, 30, and 60 min. Bioconjugation efficiency was determined by an indirect method where the spot intensity of the initial antibody amount is compared to the spot intensity after antibody bioconjugation by affiblot as given in Supporting Information Section S2.1. The densitometric analysis of the spots in Figure S-7 in the Supporting Information proved that 98% of the antibody (6 μg) was bound during 10 min of incubation and 100% during 30 and 60 min of incubation time. The efficiencies of three so-prepared MISs for SARS-CoV2 binding and RNA isolation were tested and detected by RT-qPCR.

Antibodies contain approximately 60–80 lysine residues in total, with ∼20–25 in each heavy chain and ∼10–15 in each light chain.^42^ However, not all lysines are accessible for chemical modification: some are buried within the protein’s tertiary structure, others participate in stabilizing interactions, and some are located near the antigen-binding site, where modification could impair function. In practice, only about 10–20 lysine amino acids are typically surface-exposed and reactive under standard conditions.^43^

If the bioconjugated antibody is used for immunoaffinity isolation of a viral particle as a part of a PCR-based detection system, then preserving its binding affinity is crucial. Overconjugation, for example, through excessive use of NHS esters, may impair protein function, promote aggregation or instability, and diminish the efficiency of signal amplification. Therefore, careful control of the conjugation stoichiometry is essential to maintain both functionality and assay sensitivity. The reduction of the incubation time can also influence the final immunocapture efficiency, which can lead to decreased target capture, ultimately resulting in a higher cycle threshold (Ct) value in RT-qPCR-based detection.

Each MIS lot obtained from the LIS automated and the manual procedure was divided into four 100 μg aliquots, and each aliquot was incubated with one of the four available nasopharyngeal swab samples from patients with COVID-19 diagnosis. The isolated viral particles were then lysed according to the manufacturer’s protocol and the isolated RNA was analyzed using RT-qPCR (see Experimental Procedures). The whole procedure is depicted in Figure 5.

**Figure 5:**
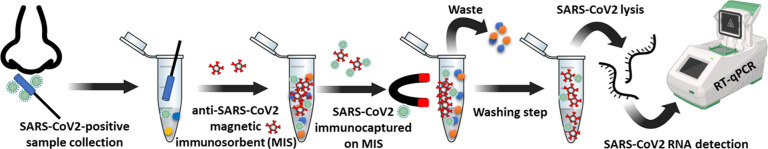
Scheme of SARS-CoV2 RNA isolation using MIS and detection using RT-qPCR. First, the nasopharyngeal SARS-CoV2 positive samples were collected in a transport medium. Then, the MIS with anti-SARS-CoV2 antibodies was added to the sample, and the viral particles were specifically immunocaptured on the surface of the MBs. The external magnet attracts the MIS to the wall of the vial, and the sample matrix was removed by pipet. Next, the viral particles were lysed, and the released RNA was analyzed on RT-qPCR. Created in BioRender. Svobodova, Z. (2025) https://BioRender.com/g55b405.

The obtained results, presented as Ct values, are summarized in Table 1. The PCR curves for each sample, along with Ct value tables and internal control data, are provided in the Supporting Information (Figures S-12 and S-13). Ct values from the LIS automated procedure (CT_LIS_) using a 60 min incubation (with a standard deviation of 0.05–0.1) were consistent with those from manually (CT_M_) prepared MIS. We calculated the delta Ct (ΔCt = CT_M_ – CT_LIS_), revealing that differences between the two methods were less than 0.5 cycles, indicating high comparability and negligible differences in Ct values. This suggests that the LIS method is a viable alternative to the manual method, offering the advantages mentioned earlier.

**Table 1:** RT-qPCR Detection of Isolated SARS-CoV2 RNA from Four Samples (S1–S4) of COVID-19-Positive Patients by Four Syntheses of MISs^a^

RT-qPCR	S1 [Ct(|ΔCt|)]	S2 [Ct(|ΔCt|)]	S3 [Ct(|ΔCt|)]	S4 [Ct(|ΔCt|)]
LIS procedure with 10 min	25.71 (1.76)	25.39 (2.37)	29.07 (2.91)	29.07 (2.33)
LIS procedure with 30 min	25.56 (1.61)	25.53 (2.51)	29.75 (3.59)	28.43 (1.69)
LIS procedure with 60 min	23.61 (0.34)	23.24 (0.22)	26.13 (0.03)	27.17 (0.43)
Manual procedure	23.95	23.02	26.16	26.74

a Ct – cycle threshold, |ΔCt| = |(CT_Manual_ – CT_LIS_)|. ΔCt < 0.5 is considered acceptable for duplicates, ΔCt = 1 means a 2-fold difference in nucleic acid quantity, and ΔCt > 1.5 means a significant reduction in nucleic acid yield or quality.

LIS-MIS synthesized with shorter incubation times of 10 and 30 min showed lower immunocapture efficiency, as reflected by ΔCt values greater than 1.5. This indicates a significant difference, with the yield of isolated nucleic acid being more than two times lower compared to both the manual MIS and the 60 min LIS-MIS. Additionally, these shorter incubation times were associated with higher Ct standard deviations (0.05–0.7), indicating increased variability. Although the indirect detection of bioconjugated antibodies on affiblot referred to almost 100% bioconjugation efficiency, we assume that the antibodies did not bind covalently due to the short time of incubation and were subsequently released. In conclusion, incubation times shorter than 1 h for LIS bioconjugation are not recommended. The 60 min LIS-MIS procedure achieved immunocapture efficiency comparable to the manual MIS, which required 2 h of incubation and manual handling. Reaching equal results in both MIS, manual, and 60 min LIS, confirmed that LIS-MIS synthesis conditions are effective in isolating limited amounts of viral RNA from the four tested samples.

In summary, the automated LIS method not only reduces processing time and minimizes manual intervention through full automation but also offers superior reproducibility in MIS recovery with a comparable immunocapture efficiency. Enhanced magnetic bead handling further highlights the LIS system as an effective tool for ligand bioconjugation on magnetic beads. Previous studies mentioned in the Introduction demonstrate that LIS systems readily integrate with downstream detection instruments, enabling on-demand preparation of MIS tailored precisely to the research requirements. This approach facilitates the generation of MIS in specific quantities and with customizable ligand densities on MB surfaces, optimizing ligand usage and enhancing reproducibility in results.

## Conclusion

The system presented in this study marks the first automated in-syringe synthesis of magnetic immunosorbents (MIS) using carbodiimide chemistry, which is the predominant method for covalent ligand conjugation on magnetic bead (MB) surfaces. This novel, automated approach generates functionalized MIS with a performance comparable to manually synthesized MIS, demonstrated here by its successful application to the analysis of COVID-19-positive nasopharyngeal samples. By addressing a critical need for automated, miniaturized production of freshly prepared MIS, the Lab-in-Syringe (LIS) system enables direct adaptation of established manual protocols with minimal parameter adjustments while substantially enhancing handling precision, MIS recovery rates, and efficiency of the overall workflow. The bioconjugation time of MBs with antibodies was reduced from 2 h needed in the manual procedure to 1 h using the automatic LIS system. However, further shortening of this process is not recommended, as MISs with 10 and 30 min bioconjugation times showed a somewhat lower yield of isolated SARS-CoV-2 RNA. This is likely due to the carbodiimide bioconjugation chemistry employed, which requires a longer duration to form a stable covalent bond as well as the fact that steric hindrance will slow down binding reaction with increasing bead coverage with antibodies. If further optimization is needed, exploring alternative bioconjugation techniques may be advisable.

Experimental results underscore the LIS system’s versatility and adaptability in bioconjugate synthesis, efficiently producing between 0.2 and 1 mg of MIS per run within a 1 mL syringe with reproducibility, which is ideal for limited or costly ligands. Additionally, small-scale synthesis offers advantages for applications requiring small MIS quantities due to stability considerations. Preliminary scalability studies indicate promising potential, with syringes up to 5 mL allowing the synthesis of 1 to 20 mg of MIS per run. The platform offers precise control over key parameters in the bioconjugation process—such as incubation duration, mixing speed, and magnetic capture strength, supporting fine-tuning for optimized bioconjugation outcomes. Notably, the system’s ease of operation and minimal training requirements were validated through trials conducted with undergraduate students.

Looking ahead, further integration of MBs and the LIS system holds significant promise. Testing a wider variety of MB sizes, syringe volumes, alternative bioconjugation techniques, and ligand types could broaden the platform’s utility across diverse applications in biomolecular assemblies and therapeutic delivery. Such advancements could also enable seamless coupling with online hyphenated analytical instruments, such as microfluidics and liquid chromatography, for enhanced sensing and analytical capabilities.

## Experimental Procedures

### Reagents and Working Material for the Manual Procedure

Sera-Mag carboxylate-modified 1 μm magnetic beads, 50 mg/mL (Cat. no. 24152105050250), were obtained from Cytiva (Buckingham, UK) and used as a 1% (w/v) suspension in PBS buffer. N-(3-(dimethylamino)­propyl)-N′-ethylcarbodiimide (EDC, Cat. no. 25952-53-8), sodium N-hydroxysulfosuccinimide (S-NHS, Cat. no. 106627-54-7), 2-(N-morpholino)­ethanesulfonic acid (MES, Cat. no. 145224-94-8), tablets of phosphate buffered saline (PBS, Cat. no. P4417-50TAB), bovine serum albumin (BSA, Cat. no. A9418), and polyclonal antimouse IgG marked with horse radish peroxidase (Cat. no. 12–349) were purchased from Sigma-Aldrich (St. Louis, MO, USA). Immun-Blot PVDF membranes (Cat. no. 1620177) were from Bio-Rad (Hercules, CA, USA). Tween20 (Cat. no. 39796.01) was from SERVA electrophoresis (Heilderberg, Germany). Mouse monoclonal antispike SARS-CoV2 antibody (Cat. no. RBD 1106, 3CV2) was purchased from HyTest (Turku, Finland). The EliGene Viral RNA/DNA FAST Isolation Kit (Cat no. 409100) was obtained from Elisabeth Pharmacon (Brno, Czech Republic), and the gb SARS-CoV2 Combi diagnostic kit that enabled the detection of SARS-CoV2 (Cat no. 3232–100) was purchased from Generi Biotech (Hradec Králové, Czech Republic). The Opti-4CN substrate solution was obtained from Bio-Rad (Hercules, CA, USA).

All solutions were prepared with ultrapure water (MQ, Milli-Q purification system, Prague, Czech Republic). The following buffers were prepared for MBs washing and bioconjugation: 0.1 mol/L MES, pH 5.0; bead storage buffer: PBS buffer, pH 7.4, with the addition of 0.1% (w/v) BSA and 0.05% (w/v) sodium azide, and affiblot assays: PBS with the addition of 0.05% (v/v) Tween 20 (PBST).

For all separations of MBs from reagents, an in-house designed magnetic separator was used that was assembled from neodymium magnets and plastic elements produced by fused deposition modeling 3D printing on a DeltaQ printer (TRILAB Group s.r.o., Hradec Králové, Czech Republic) from poly­(lactic acid) filament, which were solvent-glued with acetonitrile (see SI-1 in Supporting Information).

### LIS-Automated Bioconjugation of Antibodies to MBs

This protocol is the optimized version after all tunings mentioned in Supporting Information S1.2. The operational protocol is given in Supporting Information in Tables S-I and S-II. It started with the aspiration of the MBs suspension (400 μL corresponding to 0.4 mg MBs) that was slowly stirred as described in Figure 3. To render the highest volume reproducibility, the connecting tube to the bead reservoir was emptied by the expulsion of air just before this step. The implied dead volume (tube and syringe pump head valve) was determined visually as 53 μL with 1 mmol/L alkalinized fluorescein solution.

The aspirated MBs were washed three times with 300 μL of MES buffer for 15 s (Figure 1.III.A,B). In between, the MBs were captured on the magnetic stir bar, and the buffer solution was discharged (Figure 1.III.C,D). Activation of the functional carboxylic groups on the MBs was then executed by aspirating 200 μL of the activation reagents EDC (3.75 mg) and S-NHS (0.6 mg) from the OPI and 300 μL of MES buffer into the syringe void (Figure 1.I.G). During the subsequent 10 min incubation, intermittent stirring was performed at 1500 rpm (3 s of stirring followed by a 12 s pause) to minimize mechanical stress on the beads.

Before the discharge of the reagent solutions, 100 μL of air was aspirated. This air plug served to resuspend beads that had settled in the syringe inlet. This way, a maximum of MBs could be captured by the magnetic stir bar over 60 s. Subsequently, the liquid content of the syringe was discharged at a reduced flow rate of 5 μL/min. The MBs were then washed with 400 μL of MES buffer, for which they were resuspended by stirring activation for 15 s followed by renewed MBs capture. Afterward, 500 μL of MES buffer containing 6 μg antibodies was pipetted into the OPI while being aspirated into the syringe, and the MBs were incubated for either 10, 30, or 60 min at intermittent stirring (as in activation of MBs) followed by another washing step. During method optimization, the liquid content with the unbonded antibodies (binding fraction, BF), as well as the washing solutions, were collected for quantification via affiblot.

Finally, the so-synthesized MIS was collected in an Eppendorf vial placed in a magnetic separator. For this, they were resuspended in 400 μL MES buffer and discharged at a high flow rate of 400 μL/s under simultaneous stirring at 1500 rpm (Figure 1.III.D,E). This step was repeated four more times (2 mL collection volume) to reach the highest recovery. The buffer was then pipetted off and replaced by 1 mL of storage buffer, and the MIS was kept at 4 °C until use.

To compare different incubation times in the LIS method of immunosorbent synthesis with manual immunosorbent synthesis, three MIS batches with varying incubation times (10, 30, or 60 min) were used for SARS-CoV2 isolation. SARS-CoV2 RNA was then isolated from these samples using a manual method and detected using reverse transcription-quantitative polymerase chain reaction (RT-qPCR). After each synthesis, the tubing, OPI, and syringe were sequentially washed with MQ water and 25% ethanol and then again with MQ water by applying an automated method.

### Manually Performed Bioconjugation of Antibodies to MBs

For the manual standard procedure, a magnetic separator compatible with 1.5–2.0 mL tubes was used to attract the magnetic beads (MBs) toward the inner wall of the tube, enabling their separation from the suspension. The reagents and suspension of MBs are added or aspirated using a 1 mL pipet. After addition of the solution, the MBs are mixed in a vortex and placed back in the magnetic separator. An operator waits until the MBs are not separated; then the solution can be aspirated and replaced with another reagent. Such a process is repeated until the procedure is completed. Approximately 1 mg of magnetic beads (MBs), equivalent to 20 μL of suspension, were washed three times in a 2 mL Eppendorf vial with 1 mL of MES buffer (0.1 M, pH 5.0). The carboxyl functional groups on the MBs were activated by adding 7.5 mg of EDC and 1.25 mg of S-NHS, both dissolved in 1 mL of MES buffer, followed by a 10 min incubation on a rotator at approximately 20 rpm. After removing the activation reagents, 12 μg of anti-Spike SARS-CoV-2 antibodies, dissolved in 1 mL of MES buffer, were added to the MBs. The coupling reaction was allowed to proceed at room temperature with constant rotation for 2 h. The resulting MIS was then washed five times with MES buffer and stored at 4 °C in the storage buffer. The efficiency of bioconjugation was indirectly assessed by comparing the antibody solution before and after bioconjugation using affiblot analysis (see Supporting Information S2.1).

### Collection of Samples from COVID-19-Positive Patients

Nasopharyngeal swab samples were collected from four patients at the University Hospital in Hradec Kralove, Czech Republic. They were transported in a sample preservation solution for SARS-CoV2 virus inactivation (Jiangsu Mole Bioscience, Taizhou, China) and used to evaluate the bioconjugation efficiency in the LIS system as a proof of concept. All subjects gave their informed consent to molecular biology tests before they participated in the study. The study was carried out according to the Declaration of Helsinki and the protocol was approved by the Ethics Committee of the Charles University Hospital in Hradec Kralove, Czech Republic (project identification: COVID-19 FN HK Project A, reference number 202005 S01P/2020).

### SARS-CoV2 Immunomagnetic Separation and RNA Isolation

MBs were bioconjugated with monoclonal anti-SARS-CoV2 spike antibodies via automated and manual procedures. The MISs were used for the manual isolation of SARS-CoV2 viral particles from the nasopharyngeal samples (Figure 5). Aliquots containing 100 μg of MBs were washed three times with PBS (pH 7.4) to remove the storage buffer. Then 200 μL of sample was added and the mixture was incubated for 10 min under slow rotation. After washing them twice with PBS buffer, the RNA was isolated using EliGene Viral RNA/DNA FAST Isolation Kit according to the manufacturer’s instructions.

### SARS-CoV2 RNA Detection

Elution fractions were analyzed by RT-qPCR using a gb SARS-CoV2 Combi diagnostic kit. For analysis, 5 μL of the isolated RNA extract was added to 10 μL of the Master Mix with the SARS-CoV2 Combi Assay. The reaction was carried out on a RotorGene RG-3000A (Corbett Research, Sydney, Australia) using the following program: 10 min at 42 °C for reverse transcription and 3 min at 95 °C for initial denaturation, followed by denaturation, annealing, and elongation steps for 10 s at 95 °C and 30 s at 60 °C for a total of 45 cycles, respectively. The results were processed in Rotor-Gene 6000 software, where the cycle threshold was set to 0.1.

## Supplementary Material



## Notes

Photograph of the system, a detailed protocol from FIALab SW 5.11 for the bioconjugation of antibodies on magnetic beads (MBs), and instructions on how to clean the syringe; photographs, graphs, and tables related to the optimization of the LIS system; data on the indirect detection of IgG from its bioconjugation on MBs, including a description of the method, a figure of the PVDF membrane, and a graph showing the binding efficiency; and graphs illustrating the recovery of SeraMag beads under various conditions and the RT-qPCR curves of the data from Table 1 (PDF)

## Abbreviations

BSA: Bovine serum albumin
EDC: N-(3-(Dimethylamino)­propyl)-N′-ethylcarbodiimide
LIS: Lab-in-syringe
MB(s): Magnetic micro- and nanobead(s)
MES: 2-(N-morpholino)­ethanesulfonic acid
MIS: Magnetic immunosorbent
PBS: Phosphate-buffered saline
RT-qPCR: Reverse transcription-quantitative polymerase chain reaction
S-NHS: Sodium N-hydroxysulfosuccinimide
