# Supplementary material for: Lab-in-Syringe Automated Miniaturized Bioconjugation of Magnetic Beads with Anti-SARS-CoV2 Antibodies
Source: ACS Omega. 2025 Jul 21;10(29):31359–67. doi: 10.1021/acsomega.4c11126 (PMC12311662; doi:10.1021/acsomega.4c11126)
Supplement: Supplementary file 1 [file ao4c11126_si_001.pdf]

Supplementary information

Lab-In-Syringe automated miniaturized bioconjugation of magnetic beads with anti-SARS-CoV2 antibodies

Zuzana Svobodova[a,*], Lucie Krizova[a,b], Nikola Matejkova[c], Denisa Smela[c], Martin Beranek[d,e], Zuzana Bílkova[c], Burkhard Horstkotte[b]

[a] Charles University, Faculty of Pharmacy, Department of Biological and Medical Sciences, Hradec Kralove, Czech Republic
[b] Charles University, Faculty of Pharmacy, Department of Analytical Chemistry, Hradec Kralove, Czech Republic
[c] University of Pardubice, Faculty of Chemical Technology, Department of Biological and Biochemical Sciences, Pardubice, Czech Republic
[d] Institute of Clinical Biochemistry and Diagnostics, Charles University Hospital and Faculty of Medicine in Hradec Kralove, Hradec Kralove, Czech Republic
[e] Charles University, Faculty of Pharmacy, Department of Biochemical Sciences, Hradec Kralove, Czech Republic
*Corresponding author: svobozu@faf.cuni.cz

Content table:

1. S1.1 Lab-in-Syringe system (p. 2)
2. Figure S-1 Photograph of LIS system (p. 3)
3. Table S-1: Protocol from FIALab SW 5.11 for bioconjugation of antibodies on MBs (p. 4)
4. Table S-2: Procedure CleanSyringe (p. 6)
5. Figure S-2: 3D printed magnetic separator and open port interface (p. 7)
6. S1.2 Tuning the LIS system and operation (p. 8)
7. Figure S-3: Photographs of syringe void during the steering speed study (p. 9)
8. Figure S-4: The photographs of the syringe with the stirring bar inside (p. 9)
9. Figure S-5: Measurement of leaked MBs in various flow rates (p. 9)
10. Table S-3: Flow rates for MIS discharge from the syringe void (p. 10)
11. Table S-4: Clean volumes for MIS discharge from the syringe void (p. 10)
12. S2.1 Affiblot indirect detection of IgG (p. 11)
13. Figure S-6: Affiblot scheme (p. 12)
14. Fig. S-7: PVDF membrane from affiblot (p. 13)
15. Fig. S-8: Calibration of SeraMag MBs in PBS (p. 14)
16. Fig. S-9: Recovery and leaked MBs (0.2–1.0 mg) by absorbance (p. 14)
17. Fig. S-10: Recovery of magnetic beads (0.2–1.0 mg) (p. 15)
18. Fig. S-11: Percentages of leaked MBs in a 5 mL syringe (p. 15)
19. Fig. S-12: RT-qPCR curves with the table of Ct values in the main channel (p. 16)
20. Fig. S-13: RT-qPCR curves in the channel analyzing internal control (channel JOE) (p. 17)

S1.1 Lab-In-Syringe system

The used LIS system and different elements are depicted as photography in Fiure S-1. Connections were made from flexible FEP tubing of 0.5 mm i.d. if not stated differently. It consisted of an upside-down positioned Cavro XC12+ microsyringe pump purchased from Tecan Trading AG (Männedorf, Switzerland) featuring a 12-port ceramic head valve and being equipped with a 1 mL glass syringe of 3 cm piston length (Figure S-1.IB).

Head valve position 1 was linked to a custom-designed acrylate chamber with an approximate volume of 200 µL, produced using a Photon S stereolithographic 3D printer (Anycubic, "green resin"). This component served as an open port interface (OPI), allowing for the manual pipette-feeding of small solution volumes into the LIS system. The OPI was connected to the head valve through a short PEEK capillary (3.5 cm, 0.5 mm i.d., Figure S-1.III).

Further, the solutions were connected to head valve positions (2-10) via 10 to 30-cm-long PTFE tubing: water (2), MES buffer (3), bead suspension (4), 2 mL Eppendorf vials for bead collection (7, 8), and PBS buffer (10). Position 12 was left open for air aspiration, while position 11 served for syringe content discharge to waste. The 20 mL vial of the bead suspension, connected to position 4, was constantly stirred at ca 500 rpm to avoid bead settling. For this, a magnetic stirrer was produced from 3D printed elements and a standard computer fan (Figure 1.II) as previously described[1].

To enable solution mixing inside the syringe, a magnetic micro-stir bar (6 mm long, 2 mm in diameter) was placed inside the syringe void and driven as previously described[2,3]. In short, a driving element consisting of a plastic ring holding two neodymium magnets (5 mm diameter, 5 mm length) was placed onto the syringe barrel forcing the stir bar inside the syringe to align. This driver was turned via a rubber ring from a DC motor featured from a PWM computer ventilator[4]. The driver, a pulley wheel adapter

---

[1] Gemuh, C. V.; Horstkotte, B.; Solich, P. Lab-In-Syringe with Bead Injection Coupled Online to High-Performance Liquid Chromatography as Versatile Tool for Determination of Nonsteroidal Anti-Inflammatory Drugs in Surface Waters. *Molecules* **2021**, *26* (17). DOI: 10.3390/molecules26175358

[2] Suárez, R.; Horstkotte, B.; Cerdà, V. In-syringe magnetic stirring-assisted dispersive liquid-liquid microextraction for automation and downscaling of methylene blue active substances assay. *Talanta* **2014**, *130*, 555-560. DOI: 10.1016/j.talanta.2014.06.063

[3] Gemuh, C. V.; Macháček, M.; Solich, P.; Horstkotte, B. Renewable sorbent dispersive solid phase extraction automated by Lab-In-Syringe using magnetite-functionalized hydrophilic-lipophilic balanced sorbent coupled online to HPLC for determination of surface water contaminants. *Anal Chim Acta* **2022**, *1210*, 339874. DOI: 10.1016/j.aca.2022.339874 From

[4] Maya, F.; Horstkotte, B.; Estela, J. M.; Cerdà, V. Lab in a syringe: fully automated dispersive liquid-liquid microextraction with integrated spectrophotometric detection. *Analytical and bioanalytical chemistry* **2012**, *404* (3), 909-917. DOI: 10.1007/s00216-012-6159-4

for the motor, and support to mount the PWM motor to the syringe head valve were 3D printed. The PWM motor was relay-controlled via an auxiliary supply pin of the syringe pump and the velocity was regulated by an analog circuit. The motor movement results in rotating the magnetic field that forces the stir bar inside the syringe to turn synchronically. As an alternative to a commercial stir bar, a stronger one was produced from a stack of 3 neodymium magnets (each 2 mm long, 2 mm in diameter) which were heat-sealed into a PP tube and evaluated in comparison.

For an upscaled system, a 5 mL glass syringe was used, and the stir bar was produced alike but from 4 neodymium magnets of a total length of 12 mm, 4 mm in diameter. The syringe pump and all procedural parameters were controlled via FIALab software 5.11 (FIAlab Instrument Systems Inc., Seattle, WA, USA). The final program of the procedure is described in Supplementary Information Table. S-1 and S-2.

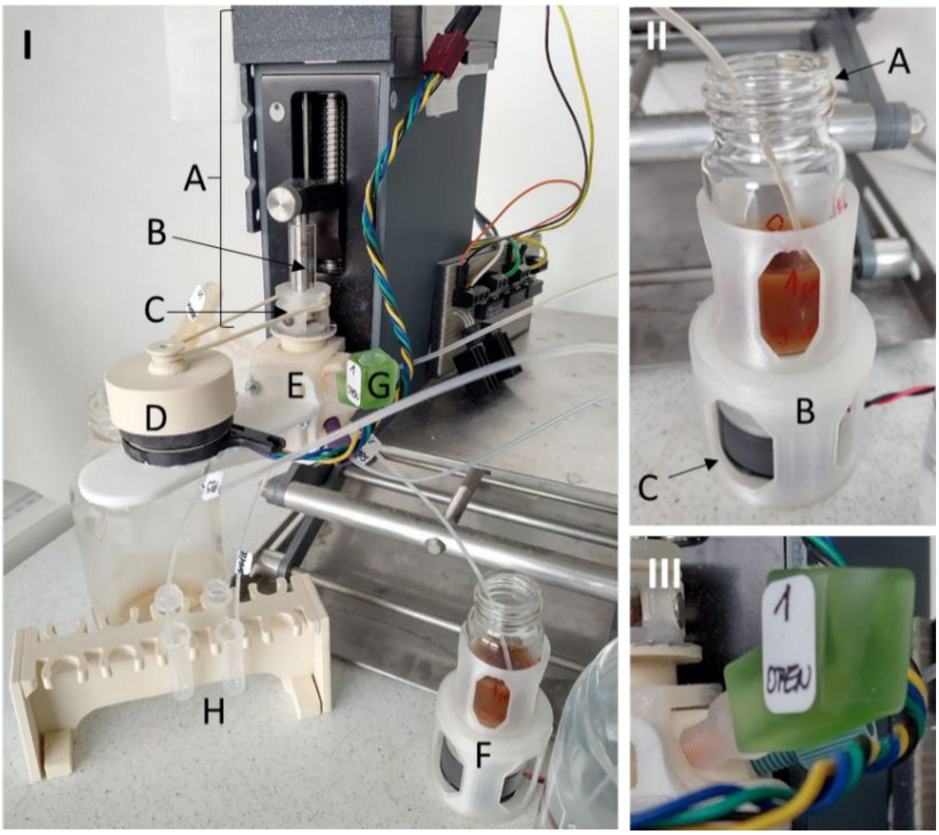

Figure S-1 Photograph of LIS system. I: A – Cavro syringe pump, B – glass syringe with PTFE piston and magnetic stirring bar inside, C – plastic driver ring with magnets surrounding the syringe, D – DC motor connected by a rubber ring to the plastic ring with magnets that can spin, E – 12-port multiposition valve, F – MBs stock solution with stirring bar, G – open port interface (3D printed), H – MIS collection into the tube placed in magnetic separator (3D printed). II: A – 10 mL glass vial with MBs stock solution with stirring bar, B – 3D printed holder, C – DC motor with two neodymium magnets that spin the stirring bar and keep the MBs in suspension. III: Detail of 3D printed open port interface for pipetting of reagents into the cylinder-shaped well (200 µL) connected to the multiposition valve. All steps were controlled by a PC with FIALab software.

Table S-1 Instruction method from FIALab software 5.11 for Lab-In-Syringe automated bioconjugation of antibodies on MBs with 30 min lasting incubation

Main step	Instruction	Comment
	CleanVol = 300<br>CleanTimes = 2<br>CleanPos = 2<br>Call procedure “CleanSyringe”	Cleaning of syringe with 300 µL water 2 times (For details see description in **Table S-2**)
Get beads	Aspirate 100 at 100 µL/s from position 12<br>Wait 1	Aspirate air to the empty connecting tube
	Dispense 100 µL at 100 µL/s to position 4<br>Wait 1	Push air to the bead tube to empty it from potentially settled beads
	Aspirate 100 µL at 100 µL/s from position 12<br>Wait 1	Aspirate air to elevate piston from surface of the liquid syringe content
	Aspirate 453 µL at 50 µL/s from position 4<br>Wait 1	Aspiration of fresh bead suspension
Wash beads 3-times	Loop Start (3)	
	Aspirate 300 µL at 100 µL/s from position 3	Aspirate MES buffer
	Activate stirring<br>Wait 15 s<br>Deactivate stirring	Washing beads by dispersion and mixing
	Aspirate 100 µL at 50 µL/s from position 12	Aspirate air to elevate beads<br>from the syringe Inlet
	Wait 60 s	Capture beads on magnetic stirring bar
	Empty syringe at 5 µL/s to position 11<br>Wait 1 s	Discharge cleaning solution at low flow rate to avoid loss of beds
	Loop End	
Activation of beads	Message “EDC + SNHS to open port interface” *	Prompt to pipette-transfer the needed amount of freshly prepared coupling agent to the open port interface
	Aspirate 200 µL at 100 µL/s100 from position 1<br>Wait 1	Aspiration of reagents EDC and SNHS from open port interface
	Activate stirring<br>Wait 1 s<br>Deactivate stirring	Activation of stirring for 1 s to mix beads with activation reagents
	Message “MES to open port interface” *	Prompt to add MES buffer to the open port interface for cleaning and completing aspiration of priorly added reagents
	Aspirate 200 µL at 100 µL/s from position 1<br>Wait 1	Aspiration of MES buffer from open port interface to clean it
	Aspirate 100 µL at 100 µL/s from position 3	Aspiration of more MES buffer for bead suspension inside the syringe
	**Loop Start (36)**	Bead incubation for 36 · ca. 16 s = 10 min
	Activate stirring<br>Wait 3 s<br>Deactivate stirring<br>Wait 12 s	Intermediate stirring to minimize bead damage
	**Loop End**	

Table S-1 Continued

Main step	Instruction	Comment
Discharge of reagents	Aspirate 100 µL at 50 µL/s from position 12	Aspirate air to suspend not-captured beads settled in the syringe inlet
	Wait 60 s	Capture beads on magnetic stirring bar
	Empty syringe at 5 µL/s to position 11<br>Wait 1 s	Empty syringe slowly avoiding bead loss
Washing of beads with MES	Aspirate 400 µL at 100 µL/s from position 3	Aspiration of MES buffer
	Activate stirring<br>Wait 15 s<br>Deactivate stirring	Suspension of the beads via stirring
	Aspirate 100 µL at 50 µL/s from position 12	Aspirate air to suspend not-captured beads settled in the syringe inlet
	Wait 60 s	Capture beads on magnetic stirring bar
	Empty syringe at 5 µL/s to position 11<br>Wait 1 s	Empty syringe slowly avoiding bead loss
Coupling of antibodies on activated beads	Message “Antibodies to open port interface” *	Prompt to pipette-transfer the needed amount of antibody solution to the open port interface
	Aspirate 200 µL at 100 µL/s from position 1<br>Wait 1	Aspiration of antibody solution from the open port interface
	Activate stirring<br>Wait 1<br>Deactivate stirring	Suspending the beads
	Aspirate 400 µL at 100 µL/s from position 3	Aspiration of MES buffer
	**Loop Start (112)**	Bead incubation for 112 · ca. 16 s = 30 min
	Activate stirring<br>Wait 3 s<br>Deactivate stirring<br>Wait 12 s	Intermediate stirring to minimize bead damage (adjustable N° of loops, 224 · ca. 16 s = 60 min)
	Loop End	
Collecting unreacted antibodies	Aspirate 100 µL at 50 µL/s from position 12	Aspirate air to suspend not-captured beads settled in the syringe inlet
	Wait 60 s	Capture beads on magnetic stirring bar
	Message new Eppendorf **	Prompt to place an Eppendorf vial to collect syringe content discharge **
	Empty syringe at 5 µL/s to position 7<br>Wait 1 s	Empty syringe
Washing of beads with MES buffer	Aspirate 400 µL at 100 µL/s from position 3	Aspiration of MES buffer
	Activate stirring<br>Wait 15 s<br>Deactivate stirring	Suspension of the beads via stirring
	Aspirate 100 µL 50 from position 12	Aspirate air to suspend not-captured beads settled in the syringe inlet
	Wait 60 s	Capture beads on magnetic stirring bar
	Message new Eppendorf **	
	Empty syringe at 5 µL/s to position 7<br>Wait 1 s	Empty syringe

Table S-1 Continued

Main step	Instruction	Comment
Collection of ready-beads	Loop Start (5) ***	
	Aspirate 400 µL at 100 µL/s from position 10	Aspirate of PBS buffer
	Activate stirring<br>Wait 5 s<br>Command (?) O8R<br>Empty syringe at 400 µL/s to position 8<br>Deactivate stirring	Suspension of beads and discharge of syringe content including beads to Eppendorf vial
	Loop End	
Syringe cleaning	CleanVol = 700<br>CleanTimes = 3<br>CleanPos = 2<br>Call procedure "CleanSyringe"	Clean syringe three times with water

* Required by the decision to use an open port interface for unstable solutions or minimal volume. Feasible automation via autosampler in case of repeated synthesis.

** Performed for evaluation of the quantity of unreacted antibodies.

*** For testing the efficiency of bead discharge, this step was repeated 5 times, which was found to be sufficient to recover approximately 99% of the initially aspirated beads.

Table S-2 Procedure "CleanSyringe"

Instruction	Comment
Loop Start (#) CleanTimes*	
Aspirate 150 µL at 200 µL/s from HV position 12<br>Go to HV CleanPos*<br>Aspirate CleanVol* µL at 100 µL/s<br>Activate stirring	Aspiration of cleaning solution while stirring
Empty syringe pump at 200 µL/s towards HV position 11<br>Deactivate stirring	Emptying syringe content to waste
Loop End	

* These variables are to define in the main program the repetition of cleaning and the cleaning solution and volume.

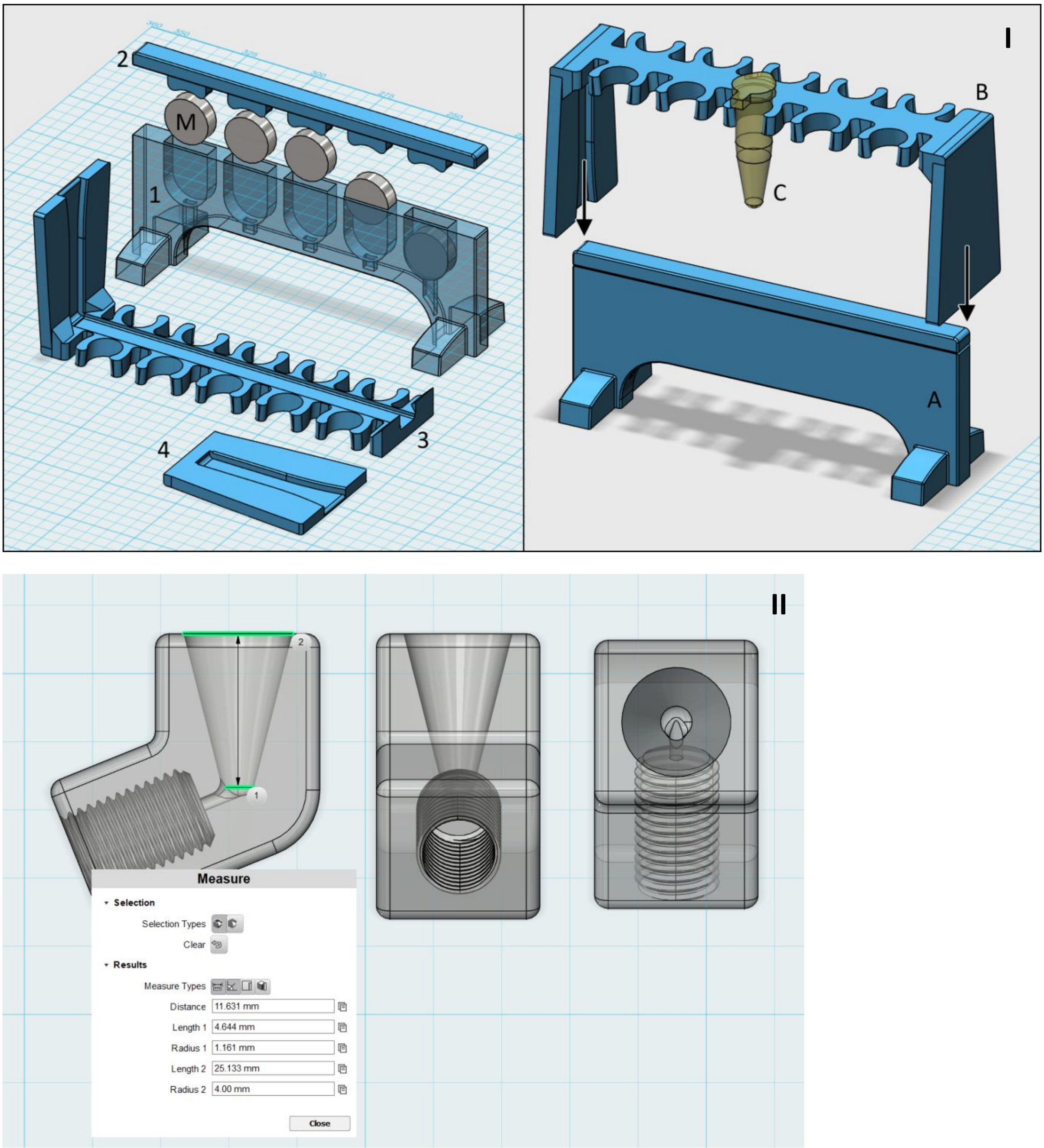

Figure S-2 I: 3D printed magnetic separator with 10 positions designed for 1.5 and 2.0 mL Eppendorf polypropylene vials. Left: separate prices for a base (1+2) containing neodymium magnets (5 mm x 15 mm in diameter) and top rack (3+twice 4). Right: assembly consisting of base containing magnets (A) and top rack (B) with indicated Eppendorf vial (C). II: 3D printed open port interface (OPI) designed for application of small volume of precious reagents (volume 200 µL max.).

S1.2 Tuning the LIS system and operation

While in the manual bioconjugation procedure consists of repeated manual steps, foremost vial manipulation, bead capture by the magnetic separator, solution switching, pipetting, or discard, bead suspending by vortexing after each magnetic separation step, and vial placement in the rotator for lasting incubations. In the LIS system, the main advantage is an all-in-one concept. The syringe void serves simultaneously as a vial, pipette, and rotator; the stir bar inside functions as a magnetic separator and vortex mixer. Additionally, it brings benefits that all steps can be optimized one by one and transformed into the software programming language commands.

The stirring speed was studied from 400 to 1700 rpm (see Supplementary Information Fig. S-3) with the aim to use minimal speed, thus applying minimal sheer forces, yet achieving efficient resuspension of all the MBs inside the syringe. By visual control, all MBs overcame the magnetic attraction of the stir bar at 1500 rpm, which was chosen as the optimal speed thereafter.

Next, we studied the stirring time for MB suspension inside the syringe. In the manual protocol, these are needed for MB activation (10 min) and bioconjugation (10 min to 2 h). Here, the typical constant agitation on a rotator in the manual procedure was replaced by intermittent stirring that proceeded as a loop of stirring and pausing to minimize mechanical stress on the beads. Stirring times from 1 to 3 s within a 15 s time window were tested. Details are given in Supplementary Information Fig. S-4. A time of 3 s stirring and 12 s waiting was finally chosen which yielded complete bead suspension.

After each washing step, solution exchange from the syringe had to be processed with minimal unintended discharge of MBs. Therefore, before liquid discharge, the MBs were allowed to be captured by the magnetic stir bar for 1 minute. Moreover, a small bubble (100 µL) was aspirated to resuspend MBs that had settled in the syringe inlet so "to give them a second chance" to be magnetically captured, which showed to be highly effective. Finally, the flow rate for solution discharge was studied in the range of 4 to 10 µL/s finding that bead loss was negligible at 5 µL/s (approximately 0.5%) while at a higher flow rate, MBs loss increased (2.1%) as shown in Supplementary Information Fig. S-5.

After complete bioconjugation of antibodies, the MIS discharge from the syringe void was fine-tuned. For this, different combinations of buffer volume (300–500 µL) and flow rates (200–400 µL/s) for solution discharge under constant stirring were tested in triplicate (see Supplementary Information Tables S-3 and S-4). For evaluation, the absorbance values of the obtained MBs suspension were compared to measurements of manually prepared controls containing the same amount of MBs (0.4 mg) and were once washed with PBS. The best option yielding nearly quantitative bead recovery (99%) was a five-fold discharge of the MBs in 400 µL PBS buffer at 400 µL/s into the collection vial.

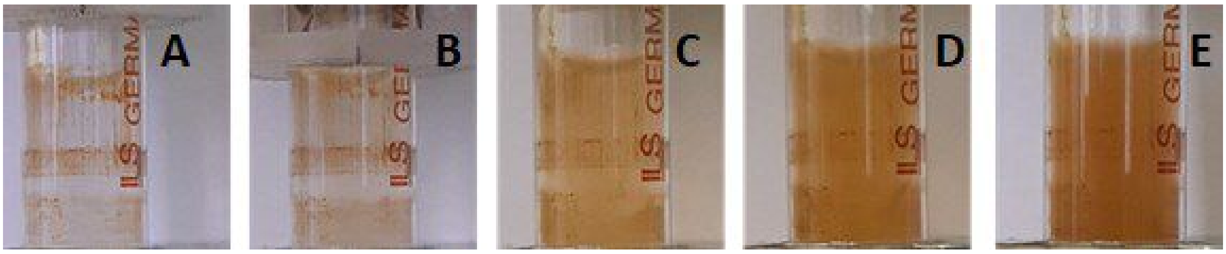

Figure S-3 Photographs of syringe void during the steering speed study: (A) 0 rpm, (B) 400 rpm, (C) 750, (D) 1100 rpm, and (E) 1500 rpm. The speed of 1700 rpm was also tested but data are not shown, the color density was the same as at 1500 rpm. The speed of 1500 rpm was selected for the experiments as it is the minimal speed when all MBs are re-suspended.

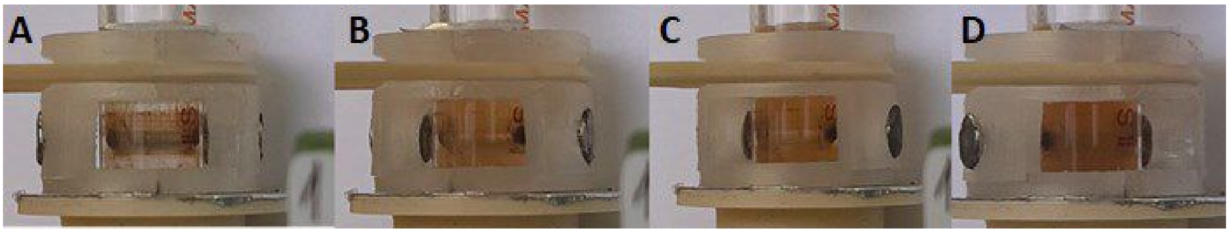

Figure S-4 The photographs of the syringe with the stirring bar inside and plastic driver ring with magnets connected to the DC motor provide mixing of the inner space of the syringe. A study of intermittent stirring was performed: (A) initial state, (B) 1 s of stirring and 14 s waiting, (C) 2 s of stirring and 13 s waiting, and (D) 3 s of stirring and 12 s waiting. The higher stirring times gave us the same results as (D). The intermittent stirring time selected for experiments was 3/12 s, where the sheering forces to MBs are minimized.

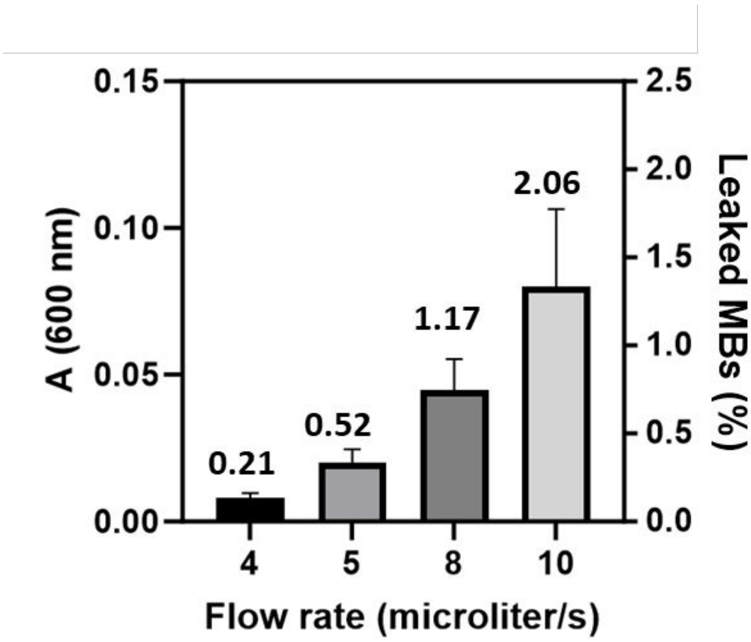

Figure S-5 Measurement of leaked MBs in various flow rates with indicated standard deviation (n = 3).

Table S-3 Effect of flow rate used for MIS discharge from the syringe void on bead recovery. Conditions: 400 µL of SeraMag MBs (0.4 mg) were aspirated into the syringe (1 mL), determination of bead quantity by absorbance at 600 nm, all values given are as absorbance units

Flow rate [µL/s]	Absorbance 600 nm			Mean	Standard deviation	Recovery* [%]
	Repetition 1	Repetition 2	Repetition 3			
200	0.712	0.877	0.804	0.798	0.068	88.37
300	0.801	0.745	0.753	0.766	0.025	84.83
400	0.782	0.827	0.818	0.809	0.019	89.92

* Signal of original MIS solution: 0.903

Table S-4 Effect of the volume of cleaning solution for MIS discharge on bead recovery (0.4 mg) with a flow rate of 400 µl/s. Determination of bead quantity by absorbance at 600 nm, all values given are as absorbance units.

Volume [µL]	Absorbance 600 nm			Mean	Standard deviation	Recovery* [%]
	Repetition 1	Repetition 2	Repetition 3			
300	0.780	0.788	0.799	0.789	0.008	87.37
400	0.793	0.782	0.803	0.793	0.009	87.82
500	0.742	0.780	0.754	0.759	0.016	84.05

* Signal of original MIS solution: 0.903

S2.1 Affiblot indirect detection of IgG

The affiblot is a dot blot-based device developed by Svobodova et al. (2020)[5] and shown schematically in Supplementary Information (Figure S-6). Briefly, the device was assembled with a polyvinylidene difluoride (PVDF) blotting membrane of 45 x 50 mm. The affiblot is a palm-sized device consisting of three parts: a lid, a support plate (middle plate), and a vacuum manifold (bottom plate), as seen in Figure S-6A (a-c). The wells in the lid are arranged in a 5 × 5 square-shaped array. Each well has a circular shape, 3 mm in diameter, and the volume might range between 10 to 100 µL, which is comparable with the standard dot blot. The upper side of the lid contains five reservoirs able to hold 1 mL of reagent, with each reservoir common for five wells in a row (Figure S-6C). The bottom side of the lid has a microfluidic drainage system (Figure S-6B) that comprises 25 short microchannels for each well – which is 200 µm wide and 50 µm deep – and five larger collector channels. The microchannels have a special crossing preventing fluid from entering the channels and well-to-well cross-talk. They also connect the well with one of the larger collector channels. Thus, the reagents can be drained from the lid reservoirs into one of the outputs connected by plastic fittings and plastic tubing with the vacuum pump (Figure S-6D,E).

The middle plate has the same hole pattern as the lid and supports the blotting membrane inserted between two perforated polyethylene (PE) foils (Figure S-6A, e-f). The PE foils protect the membrane from the fluids in the drainage channels. The bottom plate has a retention chamber for the drained fluid from the upper parts of the device. The polycarbonate plates are sealed with 2 mm thick microporous silicone (SP/16; Gumex, Straznice, Czech Republic) and are fastened with four bolts and wingnuts (Figure S-6A,i).

For our purpose, it was assembled with a methanol-activated polyvinylidene difluoride (PVDF) blotting membrane 45 x 50 mm. The membrane was subsequently equilibrated with 1 mL PBS buffer (pH 7.4) for each lid reservoir. The uploaded solutions (100 µL per well) were the antibody standard (AS) that contained the initial concentration of conjugated Ab, then the binding fraction (BF) that was collected after the conjugation step and contained the potentially unbonded Ab, and the washing fraction (W) that contained the released non-covalently bound Ab. All were pipetted into their appropriate position on the membrane. A vacuum pump was connected to the outlet at the bottom part to force the passage of the solutions through the blotting PVDF membrane.

After the antigen deposition, the membrane was blocked with 5% BSA in PBST buffer (PBS with 0.05% Tween 20; 1 mL per reservoir) and incubated for 1 h at room temperature. The blocking buffer was

[5] Svobodova, Z.; Novotny, J.; Ospalkova, B.; Slovakova, M.; Bilkova, Z.; Foret, F. Affiblot: a dot blot-based screening device for selection of reliable antibodies. *Analytical Methods* **2021**, (13), 3874-3884, 10.1039/D1AY00955A, DOI: 10.1039/D1AY00955A[5]

then drained off using the side vacuum outlet and each reservoir was filled with 1 mL washing buffer (PBST) three times and drained out using constant vacuum through the drainage microchannels and collector channels in the bottom side of the lid. Next, horseradish peroxidase (HRP)-labeled secondary anti-mouse IgG antibodies were dissolved 1:5000 in PBS-T buffer with 0.25% BSA, and 1 mL was placed per reservoir. After 1 h incubation at room temperature, the membrane was washed six times, and removed from the device. Spots on the membrane were developed in the Opti-4CN substrate solution. After 5–15 min of incubation, the membrane was washed in distilled water and photographed with a ChemiDoc™ XRS+ system (Bio-Rad Laboratories, Hercules, CA, USA). The image was processed with Image Lab™ software (Bio-Rad Laboratories, Hercules, CA, USA).

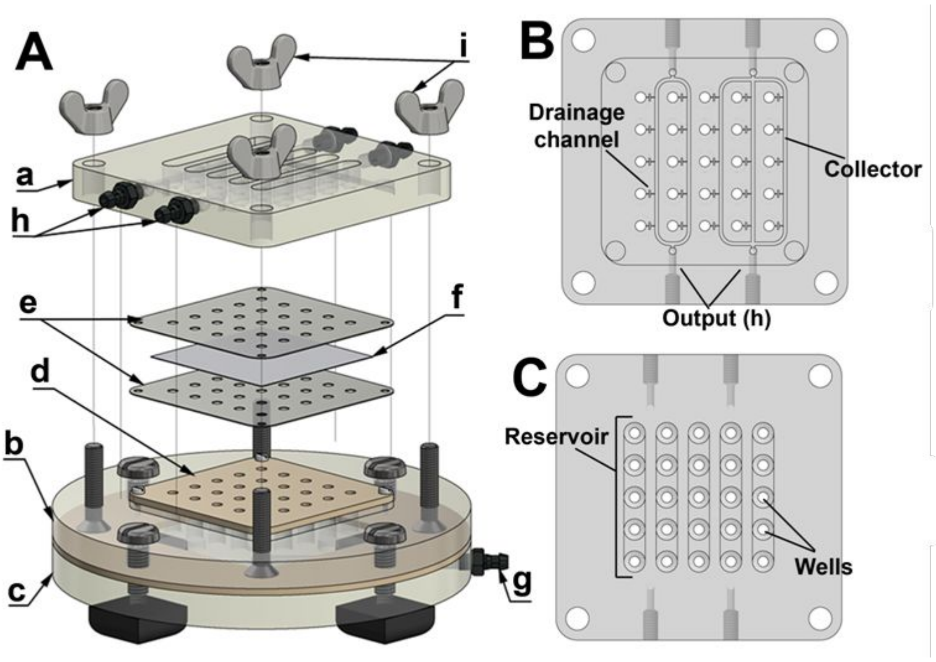

Figure S-6 Affiblot scheme a) sample template/lid, b) support plate, c) vacuum manifold, d) membrane gasket, e) PE foils, f) membrane, g) vacuum chamber outlet, h) drainage system outlets, i) wingnuts; B. Bottom side of the lid with drainage microchannels and collector channels; C. Upper side of the lid with 1 mL fluid reservoir per five wells.

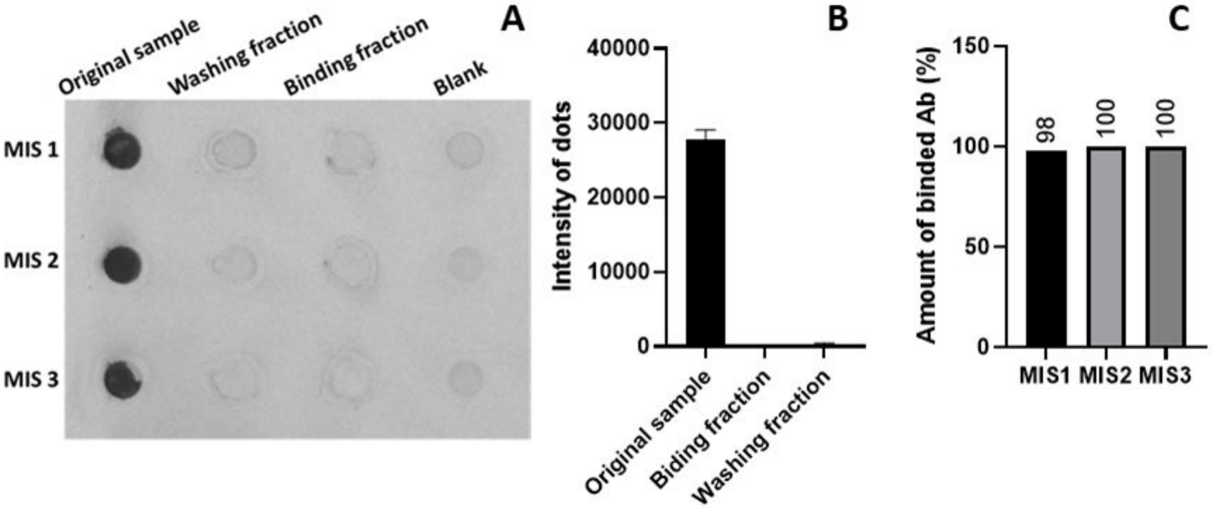

Figure S-7 PVDF membrane from affiblot (A) showing results of indirect detection of IgG from its bioconjugation on MBs. The original sample corresponds to the initial antibody concentration for each MIS aliquot. The binding fraction shows how much of the antibody stayed unbound and washing fraction shows how much of antibody was released from the MBs that were not bound covalently. Comparison of the dot intensity (B) of antibody sample before and after binding on the Sera-Mag MBs via the carbodiimide method showed that all three MISs, MIS1: LIS MIS 10 min, MIS2: LIS MIS 30 min, MIS3: LIS MIS 60 min performed efficient binding of the antibody (C) with 98-100% of 6 µg.

S3.1 Additional figures to the paper

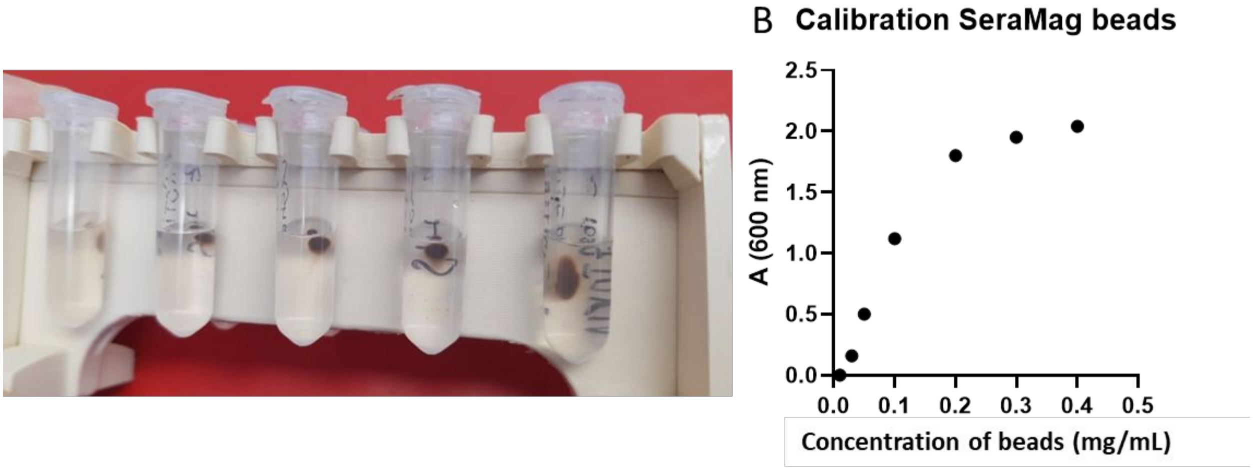

Figure S-8 Calibration of SeraMag MBs in PBS measured at 600 nm (absorbance maximum). (A) MBs in 2 mL vials in a magnetic separator before the measurement. (B) Calibration curve showing increasing absorbance with the increasing amount of MBs with linear behavior up to a concentration of 0.2 mg/mL MBs.

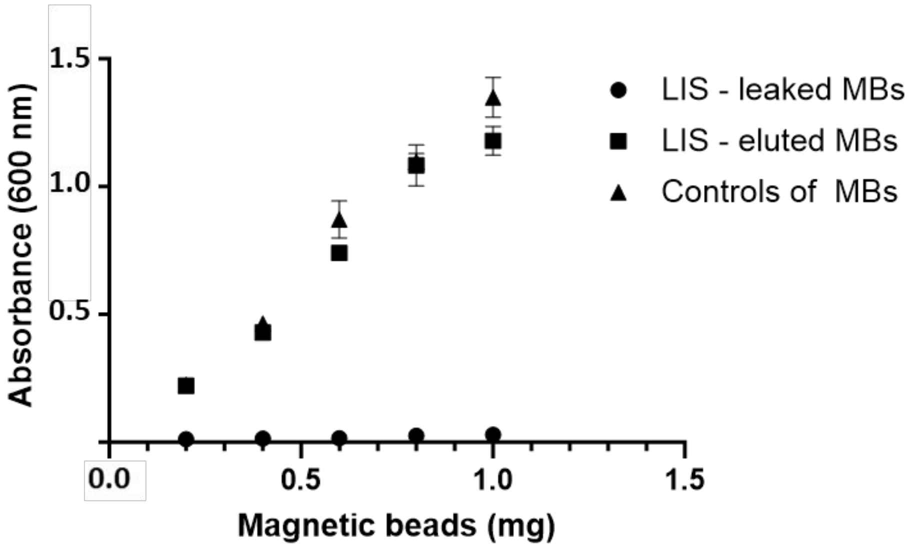

Figure S-9 Determination of recovery and leaked magnetic beads (MBs) by absorbance using absolute bead amounts of 0.2 to 1.0 mg (black squares). Found leaked MBs for each used amount in the washing step in the LIS system (black circles). Controls were prepared manually with the same volumes and bead amounts as applied in the automated procedure and washed once with PBS buffer (pH 7.4) using the magnetic separator (black triangles). Absorbance values for controls and eluted MBs were measured after their dilution 1:10 with PBS buffer. Absorbance values were measured for the recovered bead collections from a 1:10 diluted solution.

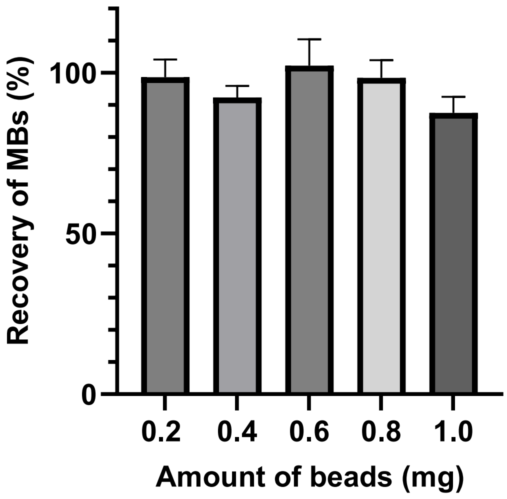

Figure S-10 Percentage of the recovery of magnetic carboxylic SeraMag beads (0.2–1.0 mg) in PBS buffer (pH 7.4) performed in triplicate. The recovery was measured as a ratio of absorbances of MBs collected after their application to the LIS system (1 mL) and calculated with reference to the control samples. The range of recovery was within 88.8–111%±3–7%.

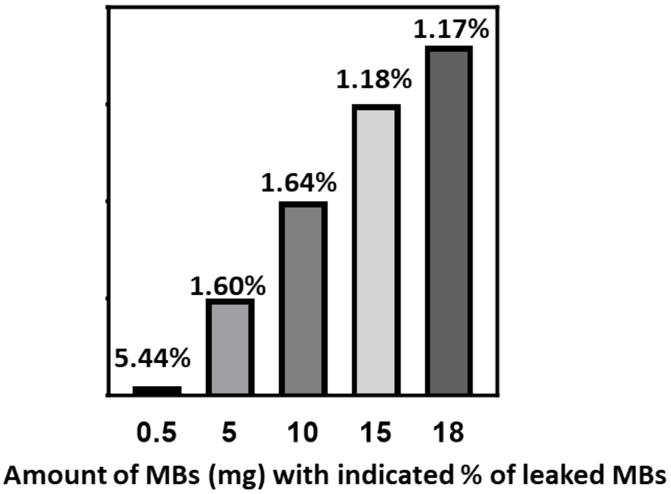

Figure S-11 Sequential accumulation of magnetic beads (MBs) in a 5 mL syringe, ranging from 0.5 to 20 mg, with the percentage of MBs leaked during the washing step indicated. The graph shows how many beads (%) leaked during one washing run for the indicated amount of MBs. Initially, 1 mg of MBs was added to the syringe, mixed, and washed once, with the washing fraction collected. Each time, this process was repeated with an additional 1 mg of MBs, ultimately accumulating 20 mg of MBs in the syringe. The washing fractions, containing MBs not retained by the magnetic stir bar, were collected, measured using a spectrophotometer at 600 nm, and expressed as a percentage of leaked MBs relative to the total amount in the syringe. The percentage of leaked MBs decreased as the MB quantity increased, from approximately 5.4% to 1.2%.

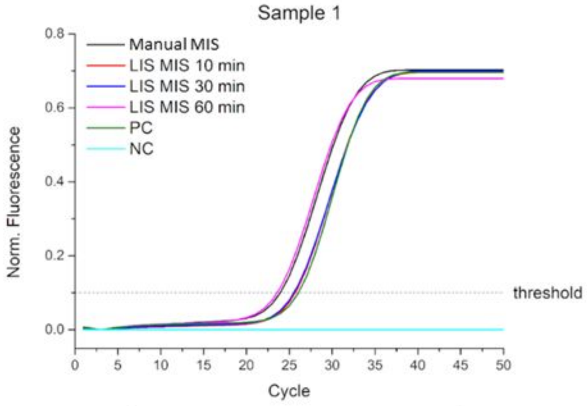

SAMPLE 1	Ct
Manual MIS	23.95
LIS MIS 10 min	25.71
LIS MIS 30 min	25.56
LIS MIS 60 min	23.61
PC	26.07

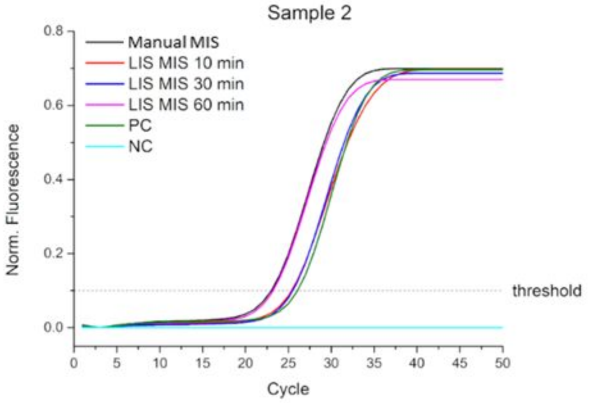

SAMPLE 2	Ct
Manual MIS	23.02
LIS MIS 10 min	25.39
LIS MIS 30 min	25.53
LIS MIS 60 min	23.24
PC	26.07

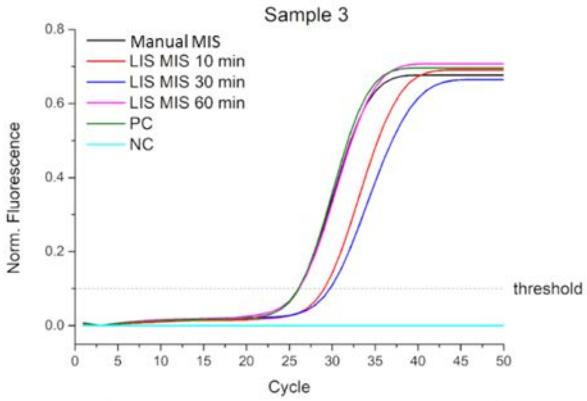

SAMPLE 3	Ct
Manual MIS	26.16
LIS MIS 10 min	29.07
LIS MIS 30 min	29.75
LIS MIS 60 min	26.13
PC	26.07

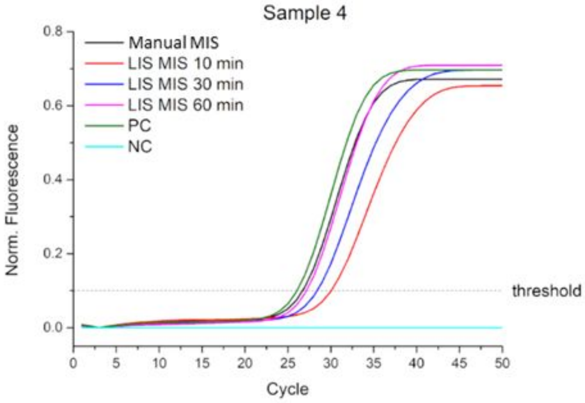

SAMPLE 4	Ct
Manual MIS	26.74
LIS MIS 10 min	30.05
LIS MIS 30 min	28.43
LIS MIS 60 min	27.17
PC	26.07

Figure S-12 RT-qPCR curves (from RotorGene RG-3000A (Corbett Research, Sydney, Australia)) with the table of Ct values in the main channel for testing different incubation times in the LIS method of immunosorbent preparation in comparison to manual immunosorbent preparation. All immunosorbents were tested on 4 positive SARS-CoV2 samples. The samples were accompanied by negative (NC) and positive control (PC) samples. The graphs were obtained directly from Rotor-Gene 6000 software.

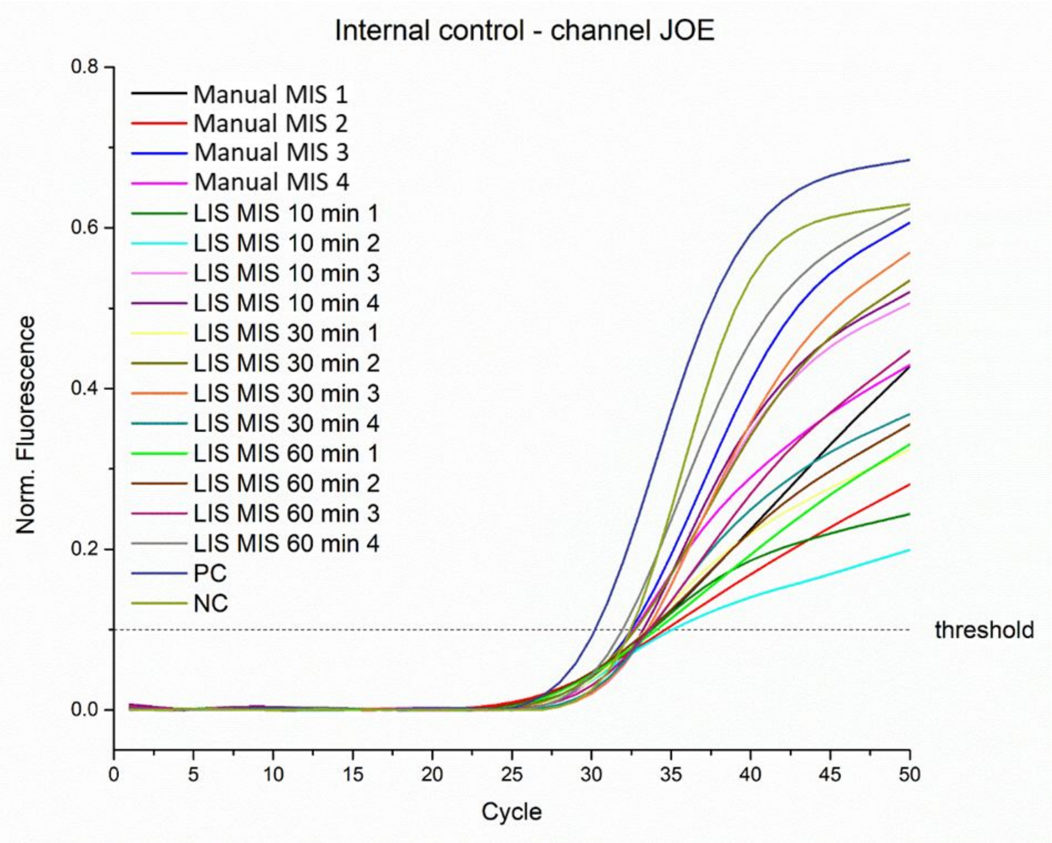

Figure S-13 RT-qPCR curves (from RotorGene RG-3000A (Corbett Research, Sydney, Australia)) in the channel analyzing internal control (channel JOE) for all tested samples and positive (PC) and negative control (NC) samples. The graphs were obtained directly from Rotor-Gene 6000 software.
